# Wind Bell-Inspired Polymeric Triboelectric Nanogenerator for Efficient Omnidirectional Wind Energy Harvesting at Extremely Low Wind Speeds

**DOI:** 10.3390/mi17080980

**Published:** 2026-08-20

**Authors:** Xichun Zheng, Haojie Li, Xue Liu, Wei Zhong, Jiwen Fang, Chong Li, Xiaohong Dong, Jiang Shao

**Affiliations:** 1 The College of Mechanical Engineering, Jiangsu University of Science and Technology, Zhenjiang 212000, China; zhengxichun@163.com (X.Z.); poovzi@163.com (H.L.); fjw617@just.edu.cn (J.F.); lichong@just.edu.cn (C.L.); 2 The College of Chemistry and Molecular Sciences, Henan University, Kaifeng 475001, China; xliu@henu.edu.cn; 3 Yangzhou Chenhua New Materials Co., Ltd., Yangzhou 225000, China; 13952581050@126.com

**Keywords:** triboelectric nanogenerators, wind energy collection, low speed wind, multidirectional wind, polymer

## Abstract

Wind energy, an abundant renewable resource, remains difficult to harness efficiently due to fluctuating speeds and unpredictable directions. In this work, we present a wind bell-inspired triboelectric nanogenerator (WB-TENG) designed for omnidirectional, variable-speed wind harvesting, utilizing layered triboelectric polymers such as polytetrafluoroethylene (PTFE), polyethylene terephthalate (PET), and polyamide (PA) to enhance energy capture performance. The developed device demonstrates the ability to generate electrical output even under extremely low wind speeds as low as 0.5 m/s. Additionally, it successfully captures wind energy from all directions within a full 360° range. Through structural optimization, the WB-TENG achieves a peak output voltage of 25.1 V and a maximum power of 3.5 μW, representing substantial improvements of 170% and 1232%, respectively, over the performance of our previous prototype. To verify its practical capability, the optimized WB-TENG is employed to power several electronic devices, including a digital watch and 50 commercial LEDs, confirming its potential for real-world energy harvesting applications. This work presents a novel and effective strategy for harnessing wind energy under dynamic environmental conditions, offering a sustainable approach for decentralized energy collection in low-speed and omnidirectional wind settings.

## 1. Introduction

Excessive reliance on fossil fuels has led to global warming, severe environmental degradation, and escalating energy concerns, drawing significant international attention [1,2,3,4]. In response, renewable and sustainable green energy has emerged as a promising strategy to address the intensifying global energy crisis [5,6,7]. Consequently, the development of environmentally friendly, efficient energy harvesting technologies is urgently required to decrease the heavy dependence on fossil fuel resources [8,9,10]. Among various alternatives, wind energy stands out as a widely accessible and clean renewable energy source, offering advantages such as abundant availability, broad geographic distribution, and minimal environmental impact [11,12,13]. In recent years, electromagnetic generators have seen extensive application in wind power generation [14,15]. However, their operation typically requires demanding starting conditions, including consistently high wind speeds and stable wind directions. In real-world environments, these requirements significantly restrict their practical use, as surface-level wind speeds are often low and wind directions highly variable and unpredictable [16,17]. Therefore, efficiently capturing and utilizing wind energy under such fluctuating conditions continues to present a considerable technological challenge.

In 2012, Wang’s team pioneered the concept of a triboelectric nanogenerator (TENG) based on Maxwell’s displacement current [18,19,20], providing a new strategy for converting low-frequency mechanical energy into electrical energy [21,22]. Owing to their advantages of lightweight characteristics, structural flexibility, and diverse material selection, polymer-based TENGs have been widely explored for harvesting mechanical energy from various environmental sources [23,24,25,26]. Particularly for wind energy harvesting, different structural designs have been developed to improve the interaction between airflow and triboelectric layers. For example, Wang et al. proposed a tension-type TENG consisting of conductive fabrics and electrospun P(VDF-TrFE) nanofibers, demonstrating the feasibility of converting wind-induced deformation into electrical output [27,28,29]. Wang and colleagues further developed a gravity-driven TENG using FEP and copper as active materials, which utilized material electronegativity differences for charge generation [30]. Feng et al. fabricated a PVDF-based polymer electrode and combined it with a Kapton triboelectric layer to investigate wind-driven energy conversion performance [31]. In addition, a flag-type TENG composed of carbon-coated PET membranes and PTFE strips was proposed to achieve wind-responsive electrical generation through membrane vibration [32]. Xu et al. developed an aerodynamic triboelectric sensor (ASTS) capable of detecting wind speeds over a broad range and identifying multiple wind directions after structural optimization [33]. Although these studies have demonstrated the potential of TENGs for wind energy harvesting, several limitations remain. Flexible membrane-based structures generally rely on airflow-induced deformation, resulting in limited output power under low wind speeds. Gravity-driven or vibration-based structures can improve mechanical response, but often require specific excitation conditions or complex structural designs. Moreover, although some devices achieve omnidirectional wind response, maintaining stable electrical output under fluctuating wind directions and variable wind speeds remains challenging [34]. Therefore, developing a simple and efficient TENG architecture capable of simultaneously achieving omnidirectional wind collection and effective low-speed wind energy conversion is still highly desirable.

**Figure 1:**
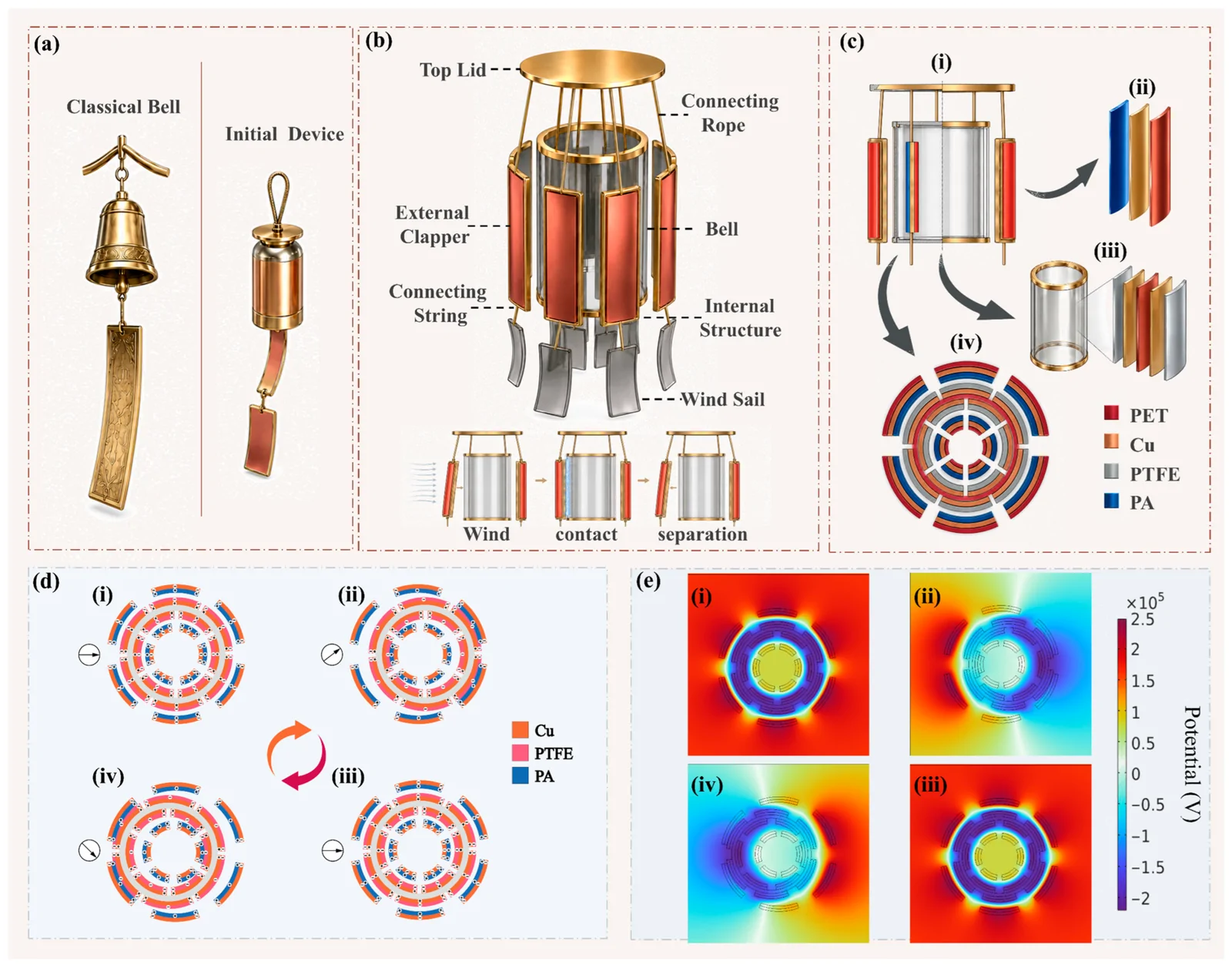
Structure and working principle of the WB-TENG. (**a**) Inspiration and diagram of the WB-TENG. (**b**) Schematic drawing of the WB-TENG. (**c**) Enlarged structural diagram of the WB-TENG: (**i**) sectional view of the main device structure, (**ii**) exploded view of the clapper structure, (**iii**) exploded view of the bell structure, and (**iv**) top view of the main device structure. (**d**) Schematic diagram of the operating principle of the WB-TENG: (**i**) equilibrium state, (**ii**) wind from the right, (**iii**) equilibrium state, (**iv**) wind from the left. (**e**) Corresponding potential distribution calculated by COMSOL Multiphysics (Version 6.0, COMSOL AB, Stockholm, Sweden) in a two-dimensional plane: (**i**) equilibrium state, (**ii**) wind from the right, (**iii**) equilibrium state, (**iv**) wind from the left.

In our earlier research, we developed a wind bell-inspired triboelectric nanogenerator (W-TENG) utilizing PTFE and PA materials, designed to efficiently capture wind energy from any direction within a complete 360° range, as shown in Figure 1a,b. Notably, this device demonstrated reliable performance at wind speeds as low as 0.5 m/s [35]. However, the output performance of the previous W-TENG is restricted by its relatively simple single-bell configuration, which provides a limited number of effective triboelectric interfaces and insufficient mechanical coupling between the wind-driven components. It delivers a modest maximum output voltage of only 9.3 V and a peak output power of 0.265 μW at a wind speed of 8.5 m/s. To overcome these limitations, this work proposes a substantially redesigned double-layer wind bell-inspired triboelectric nanogenerator (WB-TENG) featuring internal and external clapper structures. The double-layer configuration increases the effective triboelectric interaction regions and improves the conversion efficiency of wind-induced mechanical motion. Moreover, multiple structural parameters are systematically optimized to maximize the output performance. Experimental results confirm that the structurally optimized WB-TENG can convert wind energy into electrical output starting from a gentle breeze at 0.5 m/s while maintaining omnidirectional energy collection capability. Notably, the improved device achieves a maximum output voltage of 25.1 V and peak power of 3.5 μW, representing significant enhancements of 170% and 1232%, respectively, compared to our previous design. Additionally, the practical application of the WB-TENG is demonstrated by successfully powering digital watches and illuminating 50 commercial LEDs. This work offers a promising strategy for the efficient capture of omnidirectional wind energy, particularly under ultralow-wind conditions.

## 2. Materials and Methods

### 2.1. Fabrication of the WB-TENG

A schematic flowchart illustrating the fabrication process and assembly of the WB-TENG, together with the corresponding device components, is provided in Figure S1 in the Supporting Information. Fabrication of the WB-TENG: The WB-TENG “bell” is designed with an acrylic pipe (Φ = 95 mm, height 100 mm and thickness 2 mm). Twelve rectangular copper electrodes of uniform dimensions (width 50 mm, length 100 mm and thickness 0.05 mm) are arranged in equal proportion along the internal and external layers of the cylindrical shell. An equal-sized PTFE film (thickness 0.03 mm) is attached to each copper electrode to serve as the negative triboelectric layer. The WB-TENG “clappers” are produced by the method of cutting a cylindrical skeleton, which is composed of PET (thickness 0.05 mm)**.** The cylindrical framework has the same size as the acrylic pip. Each clapper has a height of 100 mm and a length of 50mm. Similarly, rectangular copper electrodes are attached to the internal surface of the external clappers and to the external surface of the internal clappers. The PA films (thickness 0.05 mm) are attached to the copper electrodes, serving as the positive triboelectric layers, as shown in Figure S2 in the Supporting Information. The “wind sails” are composed of square PC sheets (area 4 × 4 $cm^{2}$ and thickness 1 mm). The WB-TENG is equipped with a lid at the top, fabricated from PC (Φ = 100 mm and thickness 5 mm), and the six internal clappers and six external clappers are uniformly connected to the lid through connecting ropes to establish the internal and external structures. The wind-induced motion of the wind sails is transmitted to the clappers through the connecting strings, enabling the periodic contact–separation motion between the PA and PTFE triboelectric layers. The assembly of the internal and external structures within the “bell” through a process of concentric fitting results in the completion of the WB-TENG, as shown in Figure 1b.

### 2.2. Electrical Measurement

A commercial anemometer (Model GM618, BENETECH, Shenzhen, China) was used to monitor the wind speed in real time and ensure stable airflow conditions during the experiments. The electrical output signals were measured and recorded using a digital multimeter (Model 34401A, Keysight Technologies, Bayan Lepas, Malaysia) over a 5 s period. The copper electrodes of the internal and external triboelectric layers were connected to the corresponding positive and negative terminals through independent wires. The 12 output channels were subsequently connected to a rectifying bridge to convert the alternating electrical signals into direct current for subsequent electrical measurements and charging applications. A detailed schematic of the electrode connections and charge-collection pathway is provided in Figure S3.

## 3. Results and Discussion

As illustrated in Figure 1b, a Chinese wind bell inspired WB-TENG composed of a “bell,” serial internal “clappers,” serial external “clappers” as well as serial “wind sails” is proposed to effectively collect the wind energy. A WB-TENG inspired by the traditional Chinese wind bell is proposed, featuring a combination of a central “bell,” a series of internal and external “clappers,” and multiple “wind sails” designed to efficiently capture wind energy. A detailed depiction of the enlarged structural design is provided in Figure 1c. The “bell” component consists of a cylindrical shell constructed by laminating a copper electrode and a PTFE film onto the inner and outer surfaces of a PET cylindrical frame, respectively. Both internal and external “clappers” share a similar configuration, fabricated from PA films integrated with copper electrodes. The internal clappers are susp pended close to the bell’s inner surface, while the external clappers hang adjacently to its outer surface. This is achieved using ropes secured to a fixed frame at the upper edge, while the bell itself is directly attached to the same setup. Serving as the core functional elements, the wind bell structure and clappers together form the primary triboelectric interfaces that harvest wind energy and convert it into electrical output. Here, PTFE and PA polymeric films act as the complementary triboelectric polymer layers. An alternating arrangement of internal and external clappers is adopted to maximize the efficiency of wind energy capture. The “wind sails” are fabricated from square polycarbonate (PC) sheets, connected to the clappers via strings. When exposed to light breezes, these sails undergo rotational oscillation, driving the swinging motion of both internal and external clappers. Unlike a conventional single-layer structure, the alternating arrangement of the internal and external clappers increases both the number of effective PTFE–PA contact–separation interfaces and the contact frequency during wind-induced oscillation. The enhanced interaction between PA and PTFE layers promotes charge transfer during repeated contact–separation cycles, resulting in improved electrical output [36]. This movement results in continuous contact and separation between the triboelectric polymer layers, effectively transforming mechanical wind energy into electrical energy. Owing to the omnidirectional mobility of the wind sails and the balanced tumbler-like configuration, the polymer-based WB-TENG is capable of collecting wind energy from all directions.

The operational principle of the proposed WB-TENG, which relies on a combination of triboelectrification and electrostatic induction, is schematically illustrated in Figure 1d. At the initial stage Figure 1d(i), the clapper locates at its resting equilibrium position. As the wind-driven motion brings the PTFE and PA triboelectric polymer films into contact Figure 1d(ii), this triggers electron transfer from the PA layer to the PTFE surface due to the latter’s substantially higher electronegativity. During this process, a potential difference is established, causing positive charges to flow from the inner copper electrode to the outer copper electrode via an external circuit in order to restore electrostatic equilibrium. This displacement generates a transient pulse current. As the triboelectric polymer layers gradually separate and returns to its resting equilibrium position Figure 1d(iii), the induced positive charges on the outer copper electrode are fully neutralized. As the clapper swings toward the other side of bell Figure 1d(iv), the cycle reverses, producing an opposite current within the external circuit. Through this continuous contact–separation process driven by wind, mechanical energy is consistently harvested and converted into alternating current (AC) electrical output. To visualize this mechanism more intuitively, the corresponding electric potential distribution was simulated using the finite element method in COMSOL Multiphysics. The boundary condition was defined as an infinite element domain with a thickness of 5 mm. The length of the dielectric layer in the clapper is 100 mm and the width was 50 mm. The material used was PA. The length of the dielectric layer in the bell was 100 mm and the width 60 mm. The material used was PTFE. The electrode material selected was copper. Subsequently, in the material library, assign the appropriate material properties to the geometries and set the relative dielectric constants and solve it using the steady-state solver with the results shown in Figure 1e. The potential distribution contours clearly display the voltage difference established between the inner and outer electrodes, which drives charge flow through the external circuit during each oscillation cycle.

**Figure 2:**
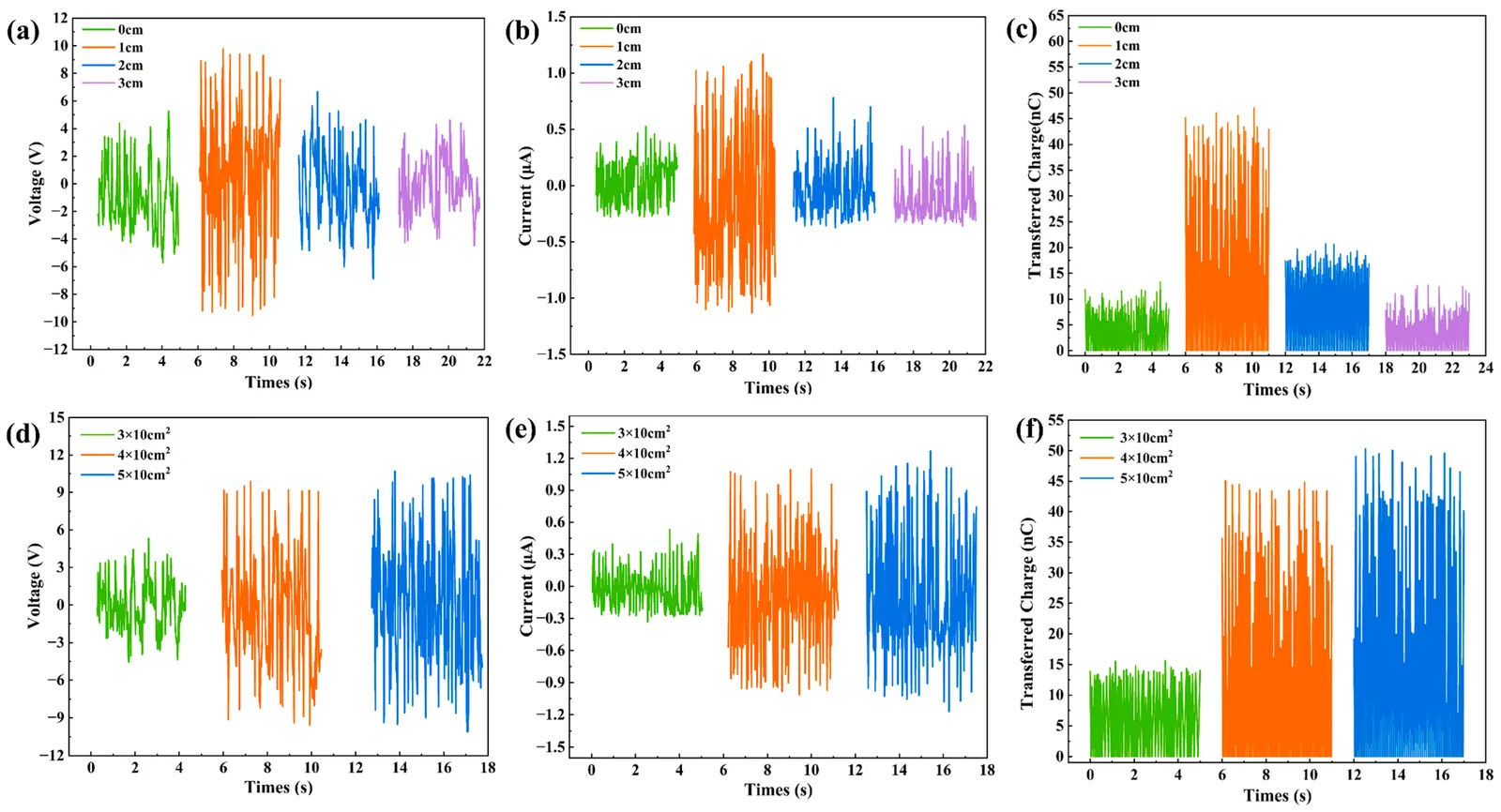
Optimizing the structural parameters of the WB-TENG. (**a**) Open-circuit voltage, (**b**) short-circuit current, and (**c**) transferred charges with different lengths of connecting string (0, 1, 2, and 3 cm). (**d**) Open-circuit voltage, (**e**) short-circuit current, and (**f**) transferred charges under different clapper sizes (3 × 10, 4 × 10, and 5 × 10 cm^2^).

To examine how structural parameters influence the output performance of the WB-TENG, a suspension platform was used to secure the device while an electric fan generated controlled wind at varying speeds and directions. The wind speed was monitored using a commercial anemometer. Focusing on the effects of connecting string length and “clapper” size, performance tests for the WB-TENG were conducted by adjusting these two parameters at a constant wind speed of 4.5 m/s and a fixed wind direction. As presented in Figure 2a–c, when the “clapper” size is set to 4 × 10 $cm^{2}$, the experimental results indicate that the electrical output of the WB-TENG improves progressively as the connecting string length increases from 0 cm to 1 cm. Beyond this point, further lengthening of the string results in a decline in performance, with the peak open-circuit voltage, current, and transferred charge (9.9 V, 1.2 μA, and 46.4 nC, respectively) achieved at a string length of 1 cm. This behavior can be attributed to the working mechanism, where the wind initially propels the “wind sail,” which then transmits force to the “clapper” via the connecting string, causing the “clapper” to oscillate and generate electricity through contact–separation motions. A direct connection between the “wind sail” and “clapper” (0 cm string length) restricts the independent motion of the “wind sail,” limiting energy conversion efficiency. Conversely, overly long strings create a looser connection, reducing the pulling force applied to the “clapper” and leading to weaker contact between the triboelectric layers, ultimately lowering the device’s electrical output. Regarding the influence of “clapper” size, as shown in Figure 2d–f, the open-circuit voltage increases as the “clapper” size enlarges, reaching a maximum of 10.4 V Similar trends are observed for both current and transferred charge. A larger “clapper” (5 × 10 $cm^{2}$) expands the stress distribution area upon contact with the triboelectric surfaces, enhancing the effective contact area and thereby improving the energy harvesting efficiency of the system.

**Figure 3:**
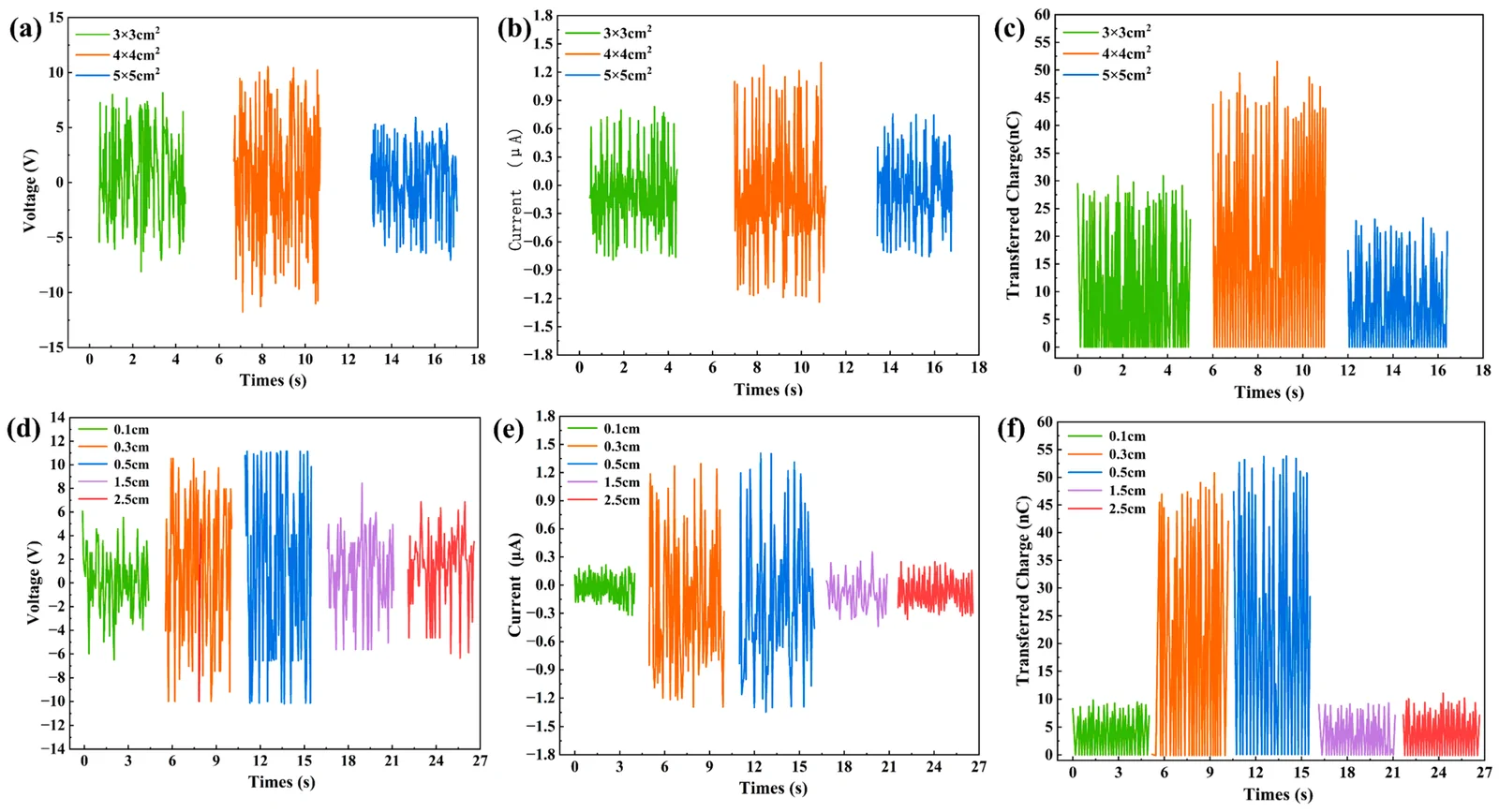
Optimizing the structural parameters of the WB-TENG. (**a**) Open-circuit voltage, (**b**) short-circuit current, and (**c**) transferred charges with different sizes of “wind sail” (3 × 3, 4 × 4, and 5 × 5 cm^2^). (**d**) Open-circuit voltage, (**e**) short-circuit current, and (**f**) transferred charges with different separation distances between triboelectric layers (0.1, 0.3, 0.5, 1.5, and 2.5 cm).

Previous studies have shown that both the size of the “wind sail” and the separation distance between triboelectric layers play critical roles in determining the output performance of TENG devices. In the developed WB-TENG, the effect of varying wind sail size on device performance is systematically examined, as depicted in Figure 3a–c. The open-circuit voltage initially increases as the sail size grows, reaching a maximum value of 10.5 V before subsequently declining. Similar trends are observed for both current and transferred charge. A larger sail enhances the stress area and, in turn, applies a greater compressive force to the triboelectric layers, increasing the effective contact area and improving the device’s energy conversion efficiency. However, when the sail size becomes excessive, the increased mass and aerodynamic resistance consume additional wind energy during oscillation, reducing the motion responsiveness of the clappers and decreasing the overall electrical output. In this work, the WB-TENG is evaluated within a wind speed range of 0–8.5 m/s, which covers the typical operating range of many practical low-speed wind environments. Considering the overall dimensions and mechanical characteristics of the device, the 4 × 4 cm^2^ wind sail achieves an optimal balance between wind capture capability and structural responsiveness. In addition, this study investigated the influence of separation distance between the triboelectric layers, examining values of 0.1 cm, 0.3 cm, 0.5 cm, 1.5 cm, and 2.5 cm, as illustrated in Figure 3d–f. Experimental results indicate that the electrical output of the WB-TENG improves progressively as the separation distance increases from 0.1 cm to 0.5 cm, peaking at 11 V, 1.3 μA, and 54.2 nC for open-circuit voltage, current, and transferred charge, respectively. Beyond this optimal spacing, further increases in separation distance leads to a noticeable decline in performance. A smaller gap ensures that the clapper’s outer size closely matches the bell’s inner diameter, creating a larger effective contact area and enhancing electrical output. However, when the separation distance is too narrow (less than 0.5 cm), it restricts the separation motion of the triboelectric layers, impeding charge transfer and consequently degrading the device’s output performance.

**Figure 4:**
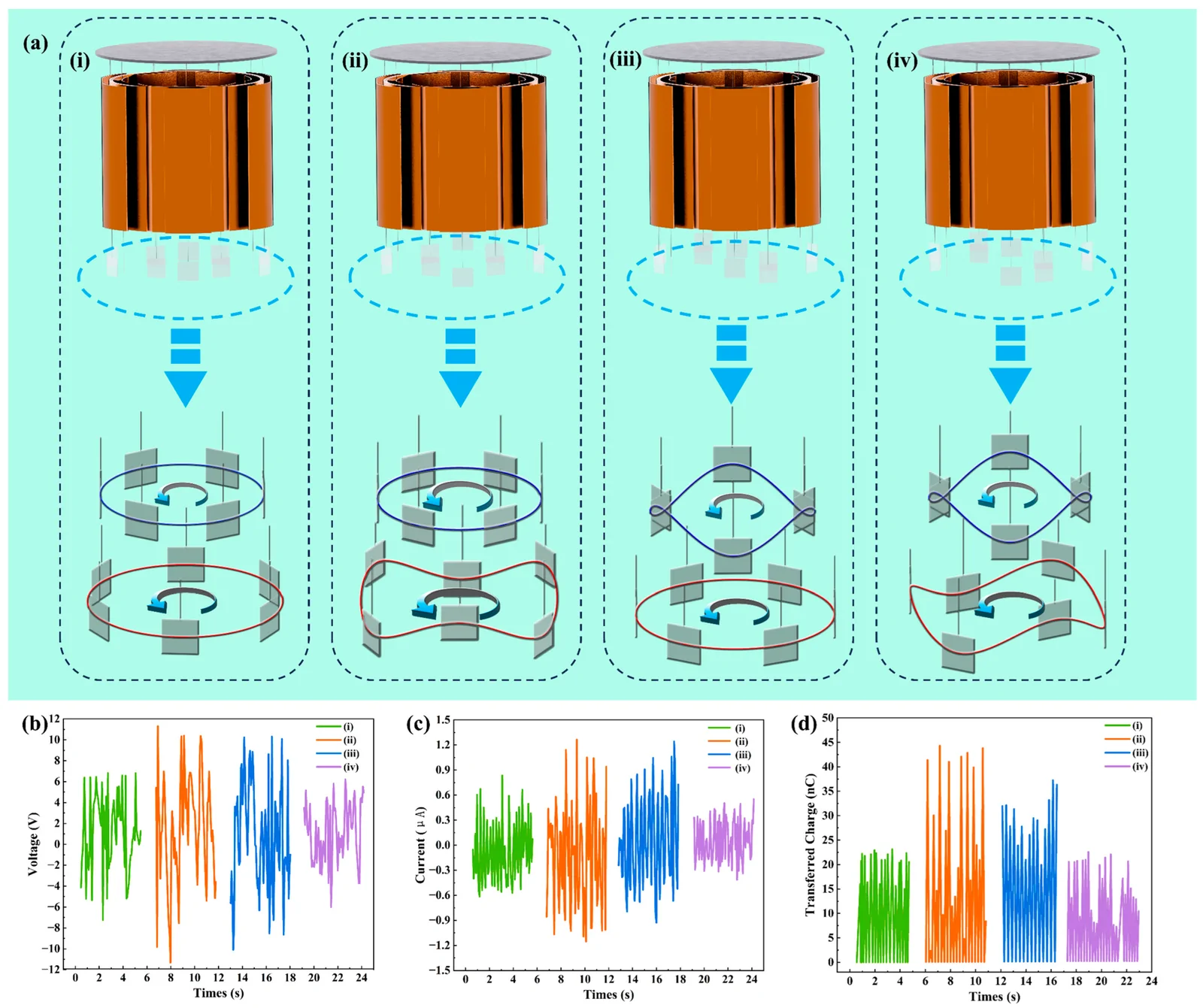
Influence on WB-TENG output power of different “wind sail” arrangements. (**a**) Schematic diagrams of four wind sail arrangements: (**i**) horizontal–horizontal configuration, (**ii**) horizontal–sine configuration, (**iii**) sine–horizontal configuration, and (**iv**) sine–cosine configuration. (**b**) Open-circuit voltage, (**c**) short-circuit current, and (**d**) transferred charges with different “wind sail” arrangements.

To gain deeper insight into how different configurations of internal and external “wind sails” affect the output performance of the WB-TENG, several samples were fabricated featuring horizontally arranged sails, sine wave-arranged sails, and cosine wave-arranged sails, as illustrated in Figure 4a(i–(v). As shown in Figure 4b–d, the WB-TENG equipped with horizontally arranged internal sails and sine wave-arranged external sails, corresponding to the configuration in Figure 4a(ii), achieves the highest electrical performance, generating a peak open-circuit voltage of 11.3 V, a current of 1.3 μA, and a transferred charge of 44.3 nC at a wind speed of 4.5 m/s. The superior performance of the horizontally arranged internal sails combined with sine wave-arranged external sails can be attributed to their optimized mechanical interaction. The internal sails provide stable motion transmission, while the sine-wave arrangement of external sails improves airflow capture and reduces interference between adjacent structures. Consequently, this configuration enhances the oscillation amplitude and increases the effective contact–separation frequency of the triboelectric layers. In contrast, when both internal and external sails are arranged horizontally, partial overlap between them can occur during operation, which reduces the efficiency of wind energy capture. Similarly, in the case of a WB-TENG featuring sine wave-arranged internal sails and cosine wave-arranged external sails, the entanglement of the connecting strings under wind conditions impairs the smooth movement of the sails, diminishing wind energy harvesting efficiency. Additionally, the configuration with horizontally arranged external sails and sine wave-arranged internal sails shows slightly lower performance than the optimal arrangement. This minor reduction may be attributed to the smaller number of external sails in that design, which limits the overall surface area available for wind energy collection.

**Figure 5:**
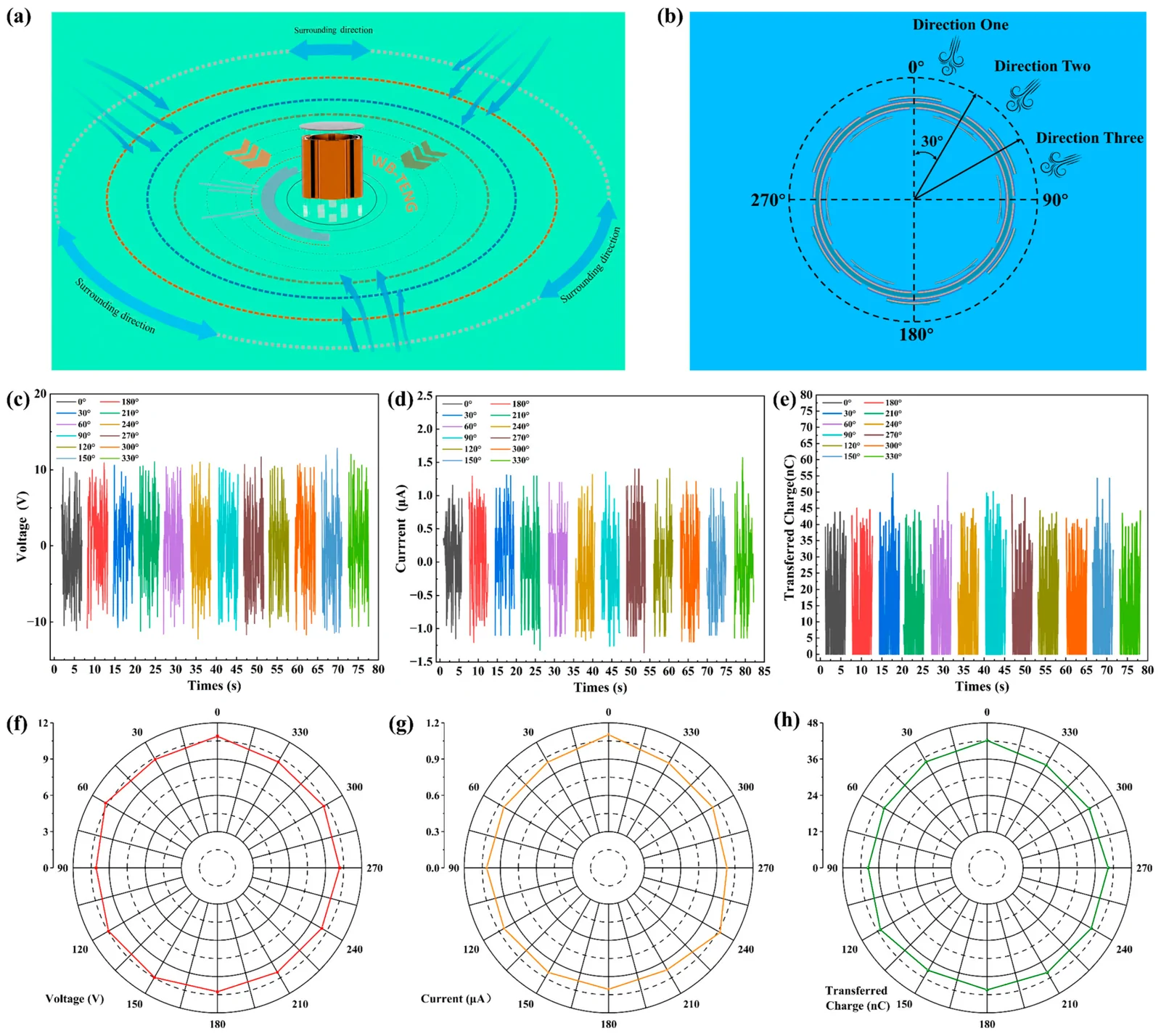
Electrical performance of the WB-TENG with different directions. (**a**) Diagram of the WB-TENG multidirectional aerodynamic test. (**b**) Schematic diagram of different wind directions and WB-TENG faces. (**c**) Open-circuit voltage, (**d**) short-circuit current, and (**e**) transferred charges on angles ranging from 0 to 360 degrees. Directional map of (**f**) open-circuit voltage, (**g**) short-circuit current, and (**h**) transferred charges.

To evaluate the omnidirectional wind energy harvesting capability of the designed WB-TENG, the device was tested under controlled conditions with varying wind directions while maintaining a constant wind speed of 4.5 m/s, as illustrated in Figure 5a,b. Measurements of open-circuit voltage, current, and transferred charge were recorded as the wind direction was incrementally adjusted in 30-degree steps, as shown in Figure 5c–h.

Unlike conventional TENGs designed for wind energy applications, it is noteworthy that the electrical output of the WB-TENG remains largely unaffected by changes in wind direction. The open-circuit voltage consistently stabilizes around 10 V across the full 0° to 360° range. This distinctive performance can be attributed to the unique structural design of the wind bell system, which allows each “wind sail” to freely reorient itself in response to external airflow. This dynamic adaptability ensures efficient wind energy collection from any direction, overcoming the limitations posed by the typically unpredictable nature of ambient wind.

**Figure 6:**
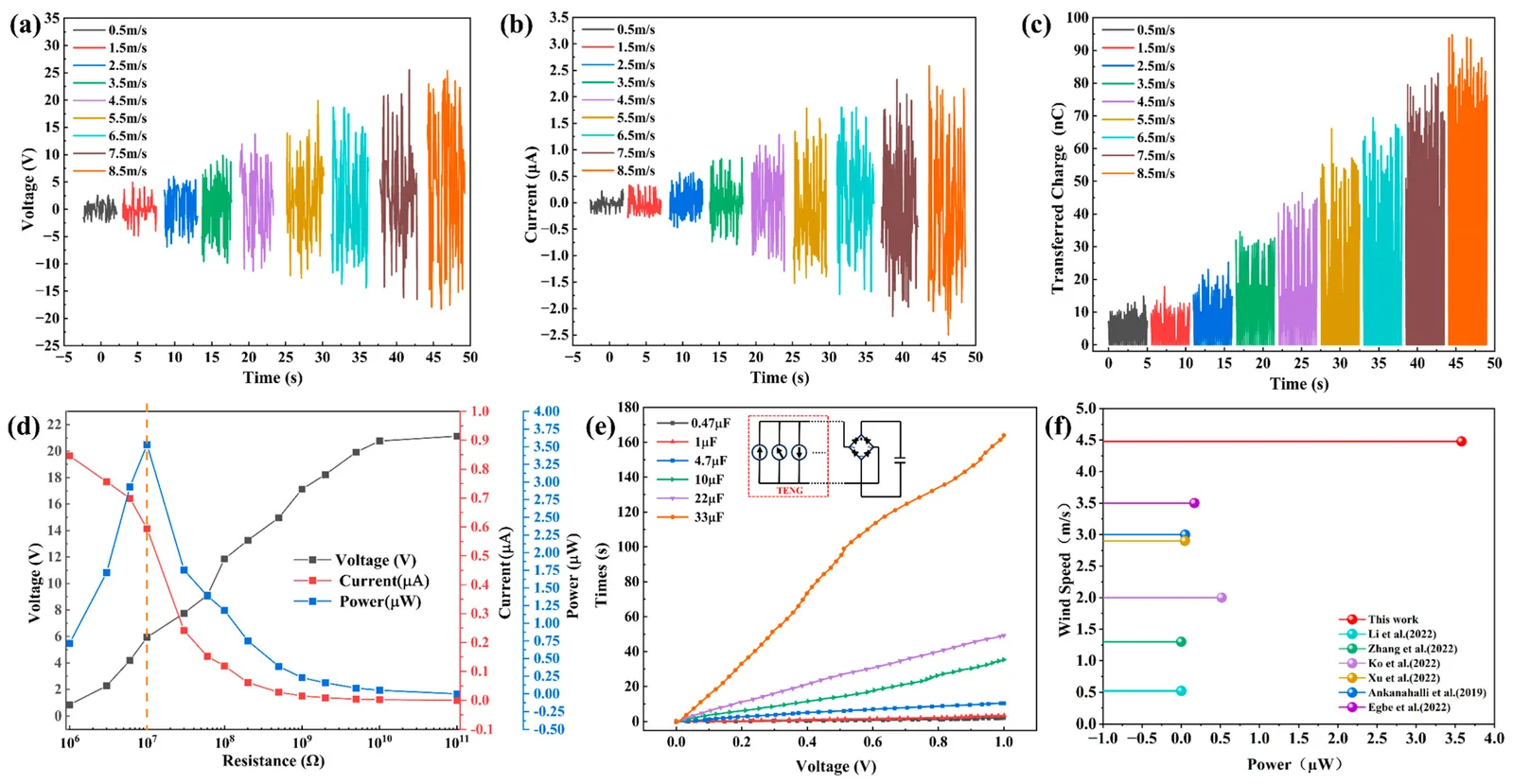
Electrical output performance of the WB-TENG. (**a**) Open-circuit voltage, (**b**) short-circuit current, and (**c**) transferred charges of the WB-TENG at wind speed of 0.5–8.5 m/s. (**d**) Amplitudes of output voltage and current and dependence of output peak power of WB-TENG under varied external load resistances at 4.5 m/s wind speed, the dashed line represents maximum output. (**e**) Voltage curves of several commercial capacitors charged by the WB-TENG harvester at a wind speed of 4.5 m/s. The inset is the charging circuit diagram. (**f**) Comparison of our results with other published results: Ref.1, the KVSM-TENG [45], Ref.2, the S-TENG [46], Ref.3, the WTENG [47], Ref.4, the ASTS [33], Ref.5, the GCP-TENG [48], and Ref.6, the H-REH [49].

In addition to evaluating wind direction effects, this study investigated how varying wind speed influences the electrical output characteristics of the WB-TENG under a fixed wind direction. As illustrated in Figure 6a–c, the device’s performance was tested over a wind speed range of 0.5 to 8.5 m/s, the operational limit of the employed electric fan. The experimental results clearly demonstrate a positive correlation between wind speed and the WB-TENG’s electrical output. At the maximum tested speed of 8.5 m/s, the device delivers a peak open-circuit voltage of 25.1 V, a short-circuit current of 2.5 μA, and a transferred charge of 94.7 nC—representing increases by factors of 9.7, 10, and 6.4, respectively, compared to those recorded at 0.5 m/s. Notably, the WB-TENG achieves an output voltage of 25.1 V at 8.5 m/s, marking a 170% improvement over our previous design. Furthermore, it is especially significant that even at an ultralow wind speed of 0.5 m/s, the device generates an output voltage of 2.6 V—to the best of our knowledge, the highest reported value for TENGs operating under such low-wind conditions. The increased wind speed enhances the contact force between the PA and PTFE membranes, promoting more intimate contact and generating a higher surface charge density. From an electrical perspective, the freestanding WB-TENG behaves similarly to a capacitor with a fixed capacitance *C*, with its output voltage and current governed by the following relationships [37,38,39,40,41]:

(1) V=Q/C,

(2) I=dQ/dt,

where *V*, Q, I, and t denote the output voltage, transferred charge, current, and time, respectively. The transferred charge is calculated by integrating the measured short-circuit current over time:

(3) Q=∫Idt,

where Q represents the transferred charge obtained during each measurement cycle. Based on these equations, it is evident that both the output voltage and current exhibit a positive linear relationship with the surface charge generated during operation. As a result, the device’s output performance improves progressively with increasing wind speed. Apart from wind speed, environmental temperature can also influence the frictional losses, charge-retention ability and dielectric properties of the polymer materials, thereby affecting the electrical output [42]. Therefore, we evaluate the overall effect of temperature on the device performance by conducting experiments at a constant wind speed of 4.5 m/s under different temperatures, as shown in Figure S4 in the Supporting Information. The device exhibits the highest output at 20 °C. From 20 to 60 °C, the output gradually decreases with increasing temperature, mainly due to enhanced charge dissipation and reduced charge retention at elevated temperatures. At 10 °C, the lower output may be associated with moisture condensation on the device surface, which can affect charge transfer. Nevertheless, the device maintains stable electrical output over the investigated temperature range.

Additionally, the output power of the WB-TENG was assessed under various external load resistances at a constant wind speed of 4.5 m/s, with the corresponding voltage, current, and power curves presented in Figure 6d. The output power was calculated using the expression [43,44]:

(4) P=V^2/R,

where *P* represents power and *R* is the external load resistance. As resistance increases, the output voltage rises while the current decreases, reaching a maximum power of 3.5 μW at a load resistance of 10 MΩ, suggesting that the internal impedance of the WB-TENG approximates this value. Relative to our earlier design, this result reflects a substantial improvement of 1232% in output performance. The device’s charging capability was further evaluated using a rectifier circuit and capacitors ranging from 0.47 μF to 33 μF, as illustrated in Figure 6e. A 10 μF capacitor charges to over 1 V within just 35 s, demonstrating the device’s rapid energy storage potential. As shown in Figure 6f, when compared to previously reported TENG systems, the proposed WB-TENG achieves noticeably higher output power, particularly at low wind speeds, confirming its excellent potential for efficient low-speed wind energy harvesting applications.

**Figure 7:**
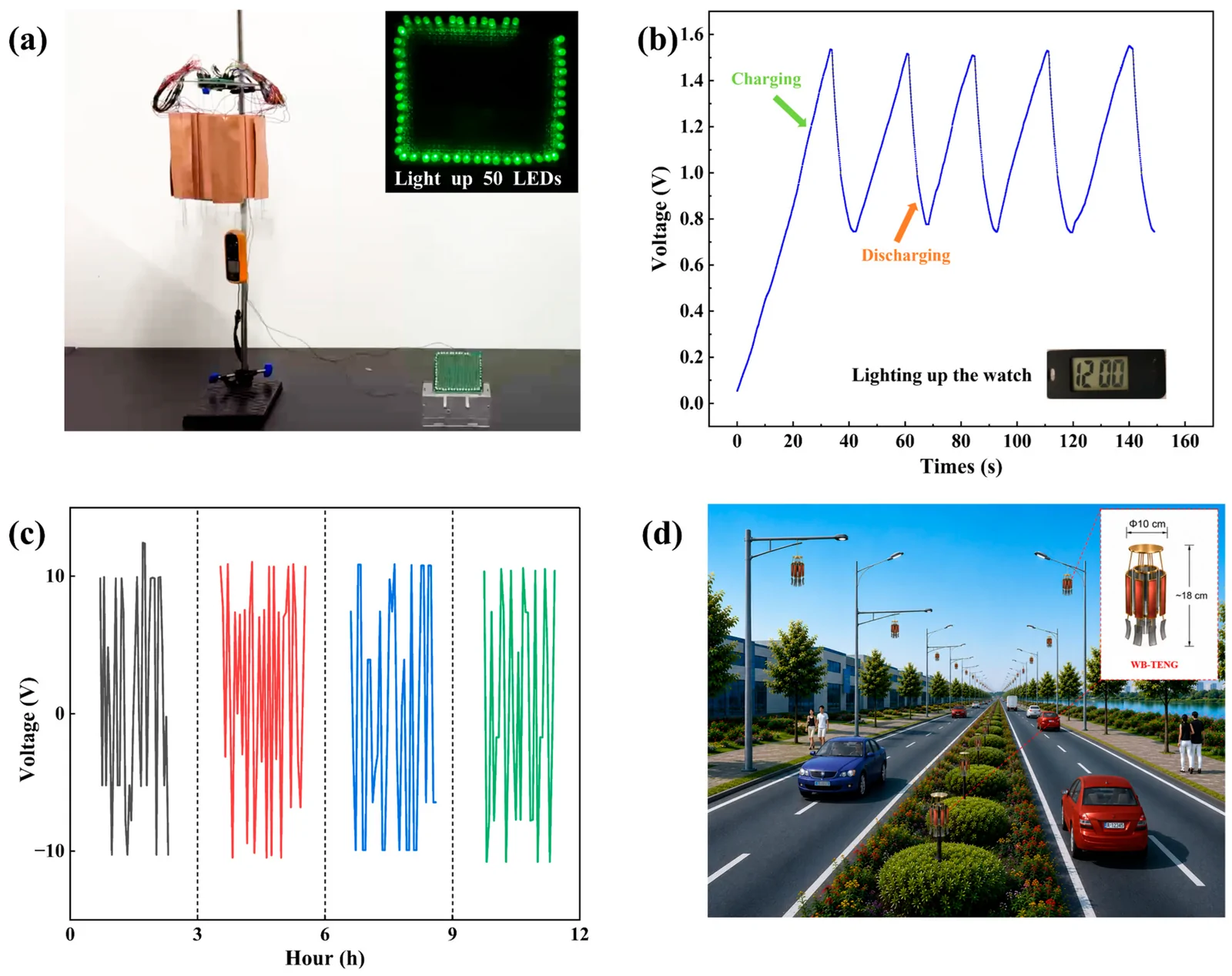
Applications of the WB-TENG. (**a**) Photograph of LEDs lit directly by the WB-TENG. (**b**) Charging voltage profile of a 10 μF capacitor for several consequent working cycles of a digital watch at a wind speed of 4.5 m/s. (**c**) Long-term operational stability test of the WB-TENG over 12 h and different colors represent different time periods. (**d**) Proposed network composed of WB-TENGs for harvesting large-scale wind energy.

A practical demonstration of the proposed WB-TENG was conducted by harvesting ambient wind energy to illuminate 50 LEDs, as shown in Figure 7a and detailed in Supplementary Video S1. Additionally, the WB-TENG, combined with a 10 μF capacitor and a bridge rectifier, was successfully utilized as a power source to operate a digital watch, as illustrated in Figure 7b and Supplementary Video S2. To assess its durability, the long-term operational stability of the WB-TENG was tested continuously for over 12 h, with no noticeable decline in performance, as presented in Figure 7c. Based on these results, we anticipate that the WB-TENG can be effectively integrated into distributed energy networks and extensively deployed in outdoor environments for scalable wind energy harvesting applications, as envisioned in Figure 7d.

## 4. Conclusions

In this work, expanding upon our earlier research, we present a WB-TENG inspired by the structure of a traditional Chinese wind bell. The uniqueness of its structure not only provides effective measures for collecting omnidirectional wind energy, but also demonstrates significant advantages in terms of the required wind speed for startup among numerous omnidirectional wind energy collectors. The device features a cylindrical “bell” multiple internal and external “clappers,” and several “wind sails,” with PTFE and PA films serving as the triboelectric materials. A detailed investigation was carried out to assess how both external wind conditions and internal structural parameters influence the electrical performance of the WB-TENG. Following systematic optimization, the final WB-TENG configuration consists of a cylindrical bell, 12 wind sails, each sized 4 × 4 $cm^{2}$, and a series of internal and external clappers sized 5 × 10 $cm^{2}$ connected by 1 cm strings. Under these optimized conditions, the device achieves peak power output of 3.5 μW, along with a maximum voltage of 25.1 V, a current of 2.5 μA, and transferred charges of 94.7 nC. Remarkably, the enhanced WB-TENG delivers a maximum voltage of 25.1 V and peak power of 3.5 μW, representing impressive improvements of 170% and 1232%, respectively, over the previous iteration. Experimental results further verify that the WB-TENG is capable of omnidirectional wind energy collection across the full 0°–360° range without any noticeable decline in performance. Particularly noteworthy is the device’s exceptional capability to harvest energy under ultralow-wind-speed conditions, producing an output voltage of 2.5 V at a mere 0.5 m/s breeze—one of the highest reported values for TENGs operating in such environments. Additionally, practical demonstrations confirm the WB-TENG’s capacity to power a digital watch and illuminate 50 commercial LEDs, underscoring its potential as a clean, sustainable solution for distributed wind energy harvesting applications.

## Data Availability

The data that support the findings of this study are available from the corresponding author upon reasonable request.

## Supplementary Materials

The following supporting information can be downloaded at https://www.mdpi.com/article/10.3390/mi17080980/s1. Figure S1: Schematic illustration of the fabrication and assembly process of the WB-TENG; Figure S2: Structural assembly and electrical connection diagram of the WB-TENG; Figure S3: Schematic illustration of the electrical connection and charge collection pathway of the WB-TENG; Figure S4: Electrical performance of WB-TENG under different environmental temperature; Video S1: Wind speed 4.5 m/s LEDs; Video S2: Wind speed 4.5 m/s Digital watch.
