# Supplementary material for: Wind Bell-Inspired Polymeric Triboelectric Nanogenerator for Efficient Omnidirectional Wind Energy Harvesting at Extremely Low Wind Speeds
Source: Micromachines (Basel). 2026 Aug 20;17(8):980. doi: 10.3390/mi17080980 (PMC13515556; doi:10.3390/mi17080980)
Supplement: Supplementary file 1 [file micromachines-17-00980-s001.zip › micromachines-4435776-supplementary.pdf]

Supporting information

The Wind Bell-Inspired Polymeric Triboelectric Nano-generator for Efficient Omnidirectional Wind Energy Harvesting at Extremely Low Wind Speeds

Xichun Zheng [1,†], Haojie Li [1,†], Xue Liu [2], Wei Zhong [1,*], Jiwen Fang [1], Chong Li [1], Xiaohong Dong [3] and Jiang Shao [1,*]

This file includes:

Figure S1. Schematic illustration of the fabrication and assembly process of the WB-TENG.

Figure S2. Structural assembly and electrical connection diagram of the WB-TENG.

Figure S3. Schematic illustration of the electrical connection and charge collection pathway of the WB-TENG.

Figure S4. Electrical performance of WB-TENG under different environmental temperature.

1 The College of Mechanical Engineering, Jiangsu University of Science and Technology, Zhenjiang 212000, China; jiangshao@just.edu.cn

2 The College of Chemistry and Molecular Sciences, Henan University, Kaifeng 475001, China; xliu@henu.edu.cn

3 Yangzhou Chenhua New Materials Co., Ltd. Yangzhou 225000, China; 13952581050@126.com

* Correspondence: zhongwei@just.edu.cn; jiangshao@just.edu.cn

† These authors contributed equally to this work.

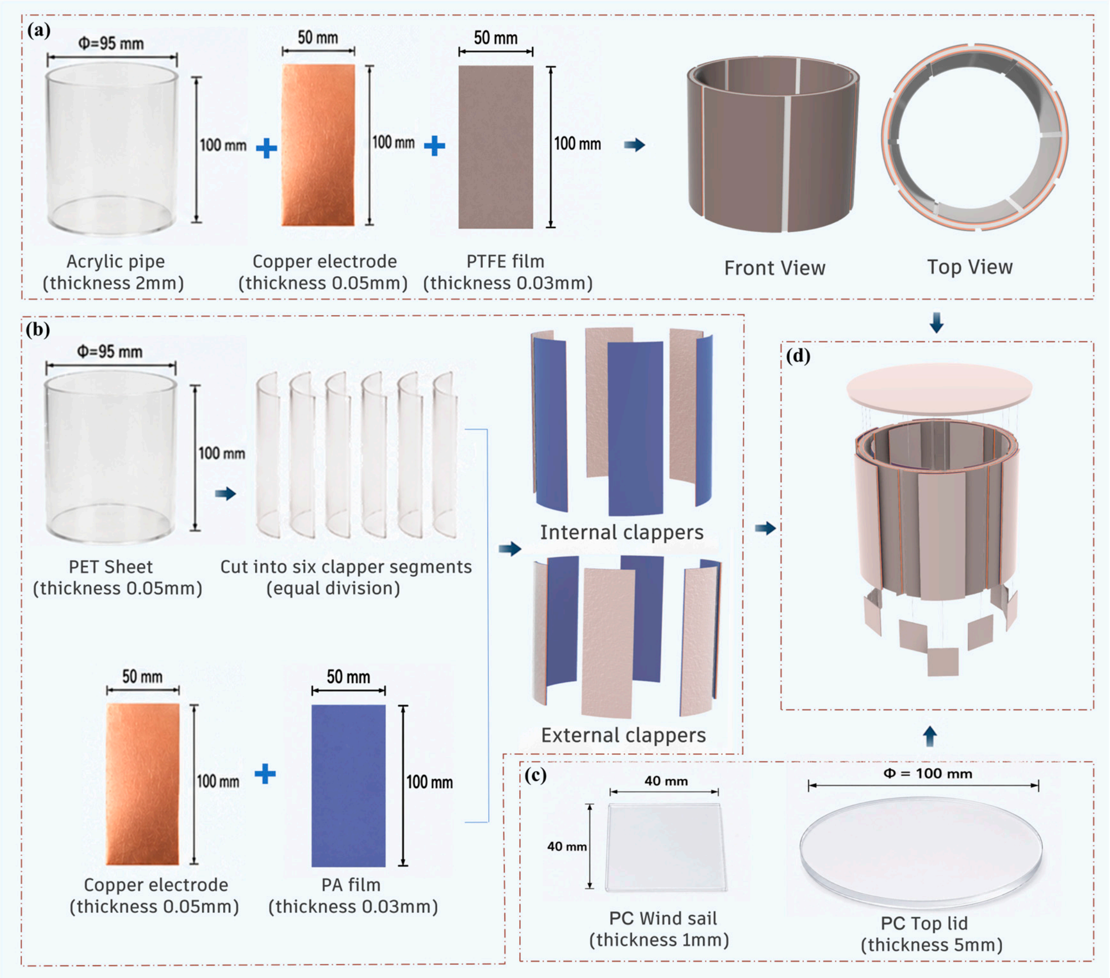

Figure S1. Schematic illustration of the fabrication and assembly process of the WB-TENG.

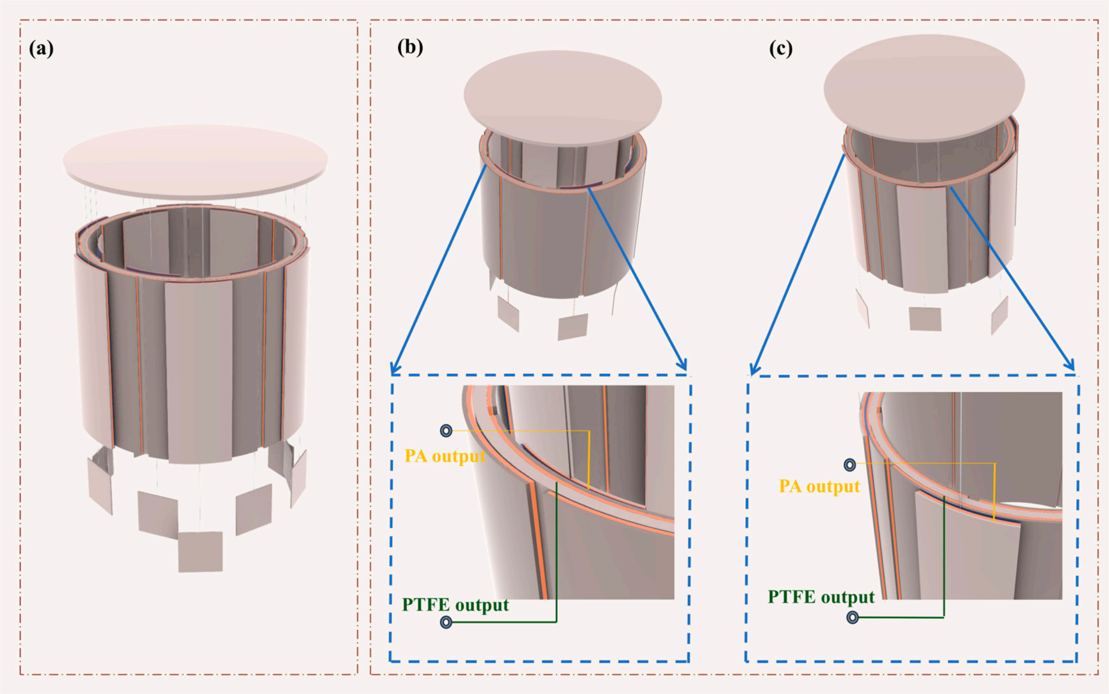

Figure S2. Structural assembly and electrical connection diagram of the WB-TENG. (a) The 1:1 scale model of the WB-TENG. (b) Schematic diagram of the electrical connection and charge collection process for the internal clappers. (c) Schematic diagram of the electrical connection and charge collection process for the external clappers.

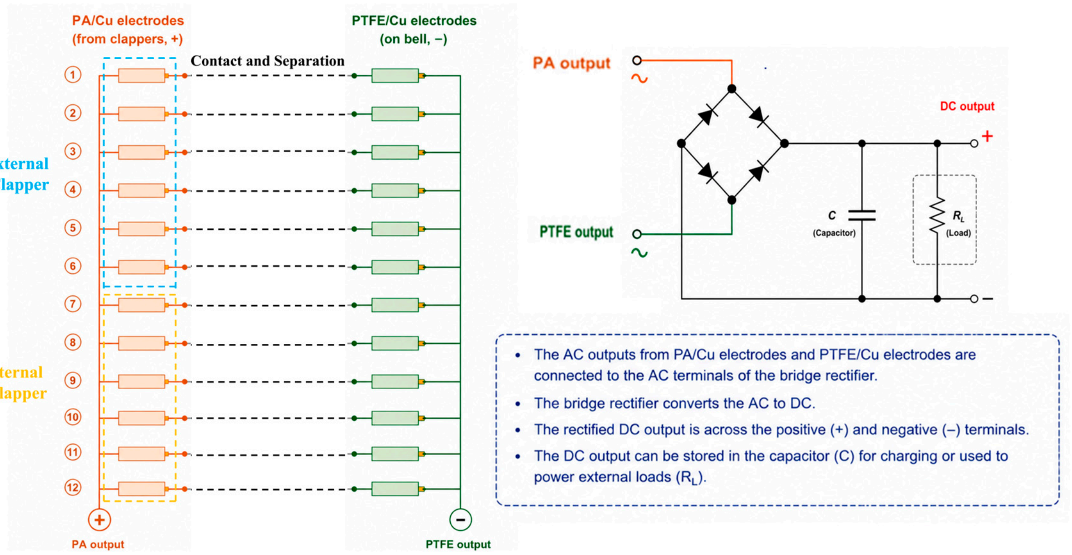

Figure S3. Schematic illustration of the electrical connection and charge collection pathway of the WB-TENG.

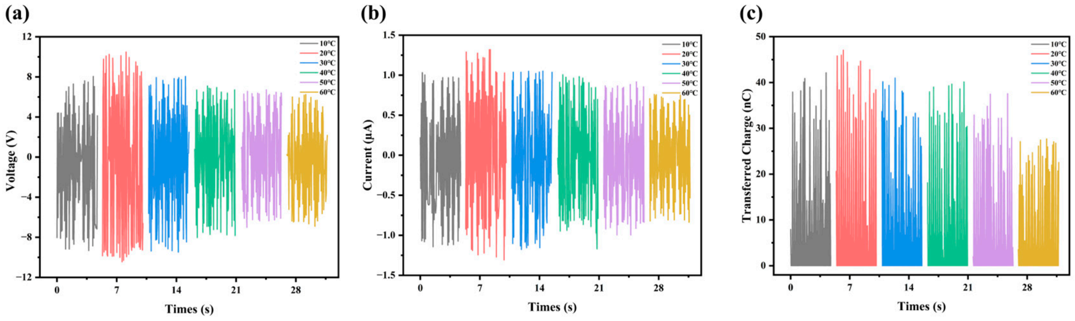

Figure S4. Electrical performance of WB-TENG under different environmental temperature. (a) Open circuit voltage, (b) short circuit current, and (c) transferred charge.
